# Autophagy and mTOR signaling during intervertebral disc aging and degeneration

**DOI:** 10.1002/jsp2.1082

**Published:** 2020-02-18

**Authors:** Takashi Yurube, Masaaki Ito, Yuji Kakiuchi, Ryosuke Kuroda, Kenichiro Kakutani

**Affiliations:** ^1^ Department of Orthopaedic Surgery Kobe University Graduate School of Medicine Kobe Japan

**Keywords:** aging, autophagy, disc degeneration, intervertebral disc, mTOR signaling, spine

## Abstract

Degenerative disc disease is a highly prevalent, global health problem that represents the primary cause of back pain and is associated with neurological disorders, including radiculopathy, myelopathy, and paralysis, resulting in worker disability and socioeconomic burdens. The intervertebral disc is the largest avascular organ in the body, and degeneration is suspected to be linked to nutritional deficiencies. Autophagy, the process through which cells self‐digest and recycle damaged components, is an important cell survival mechanism under stress conditions, especially nutrient deprivation. Autophagy is negatively controlled by the mammalian target of rapamycin (mTOR) signaling pathway. mTOR is a serine/threonine kinase that detects nutrient availability to trigger the activation of cell growth and protein synthesis pathways. Thus, resident disc cells may utilize autophagy and mTOR signaling to cope with harsh low‐nutrient conditions, such as low glucose, low oxygen, and low pH. We performed rabbit and human disc cell and tissue studies to elucidate the involvement and roles played by autophagy and mTOR signaling in the intervertebral disc. In vitro serum and nutrient deprivation studies resulted in decreased disc cell proliferation and metabolic activity and increased apoptosis and senescence, in addition to increased autophagy. The selective RNA interference‐mediated and pharmacological inhibition of mTOR complex 1 (mTORC1) was protective against inflammation‐induced disc cellular apoptosis, senescence, and extracellular matrix catabolism, through the induction of autophagy and the activation of the Akt‐signaling network. Although temsirolimus, a rapamycin derivative with improved water solubility, was the most effective mTORC1 inhibitor tested, dual mTOR inhibitors, capable of blocking multiple mTOR complexes, did not rescue disc cells. In vivo, high levels of mTOR‐signaling molecule expression and phosphorylation were observed in human intermediately degenerated discs and decreased with age. A mechanistic understanding of autophagy and mTOR signaling can provide a basis for the development of biological therapies to treat degenerative disc disease.

## Graphical abstract

Schematic illustration of disc cellular autophagy. Under stress conditions, e.g. nutrient deprivation, the mammalian target of rapamycin (mTOR), a signal integrator that detects nutrients to signal the execution of cell growth and division, is suppressed, which initiates autophagy through the activation of autophagy‐related (Atg) genes and proteins. High‐mobility group box 1 (HMGB1), which is involved in stress response, translocates from the nucleus to the cytoplasm and directly interacts with Beclin1 (Atg6 homolog). The Beclin1–class‐III phosphatidylinositol 3‐kinase (PI3K) complex initiates autophagosome formation by developing the isolation membrane. Autophagosome maturation is completed by the growth and closure of the isolation membrane, driven by the conjugation of phosphatidylethanolamine with light chain 3 (LC3) (Atg8 homolog), leading to the formation of the autophagosome‐membrane‐bound form LC3‐II. Then, p62/sequestosome 1 (p62/SQSTM1) and p62/SQSTM1‐bound polyubiquitinated proteins become incorporated into completed autophagosomes. The completed autophagosome fuses with the lysosome to form the autolysosome (which can be inhibited by chloroquine), where the enclosed cargo is degraded, and its constituents are released and recycled. Understanding of autophagy requires monitoring this dynamic, multi‐step process of autophagic flux. In our previous time‐course observational study, the graded supply of serum and nutrients decreased proliferation and metabolic activity and increased autophagy, apoptosis, and senescence in rabbit disc annulus fibrosus cells.

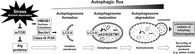

## 1 INTRODUCTION

### 1.1 Socioeconomic impacts, symptoms, and treatments of intervertebral disc disease

Back pain is a global health problem, with a lifetime prevalence of 70% to 85%,^1^ resulting in a socioeconomic burden of $102.0 billion/year in the US.^2^ The causes of back pain are multifactorial; however, a large‐scale twin study identified intervertebral disc degeneration to be an independent cause of back pain.^3^ Furthermore, disc degeneration can also present as neurological disorders, including radicular pain, numbness, muscle weakness, and paralysis.^4^ Despite the successful outcomes of conservative treatment protocols for disc disease, using medication and physiotherapy,^5^ nonresponders must undergo surgery.^6^ Current surgical interventions consist primarily of symptomatic disc excision, resulting in the loss of function, immobilization, and potential additional complications, due to altered biomechanics.^7^ Therefore, the development of new biological therapies to treat disc degeneration is an urgent issue.

### 1.2 Pathophysiology of the intervertebral disc

The intervertebral disc has a complex morphological structure, with the nucleus pulposus (NP) encapsulated by the annulus fibrosus (AF) and endplates.^4^ Although the AF originates from the mesenchyme,^8^, ^9^ the NP comes from the notochord.^10^ Notochordal cells only exist during the first 10 years of human life and are subsequently replaced by non‐notochordal, chondrocyte‐like cells of unknown provenance.^8^, ^9^ A more recent article has found that chondrocyte‐like cells are derived from disc NP cells during degeneration, the phenotype of which represents a terminal stage of differentiation preceding the loss of NP cells and disc collapse.^11^ Notochordal cell‐conditioned medium protects non‐notochordal cells from apoptosis and inflammation.^12^ In addition, notochordal cells produce larger amounts of proteoglycans than non‐notochordal cells^13^ and can stimulate non‐notochordal cells to produce proteoglycans.^14^

The collagenous, laminar AF surrounds the central, gelatinous NP, maintaining the pressurization of the NP, providing support during compressive loading, and facilitating multidimensional spinal movement.^15^ Disc AF and NP cells have chondrocytic phenotypes,^16^ producing matrix components, including proteoglycans (principally aggrecan) and collagens (predominantly types I and II in the AF and NP, respectively).^4^ Matrix metabolism is regulated by the balance between catabolic enzymes, matrix metalloproteinases (MMPs) and disintegrins and metalloproteinases with thrombospondin motifs (ADAMTSs), and anti‐catabolic inhibitors, such as tissue inhibitors of metalloproteinases (TIMPs).^17^ Increased MMP and ADAMTS levels, relative to TIMP levels, have been observed in discs during human clinical^18^, ^19^, ^20^ and rodent experimental degeneration.^21^, ^22^, ^23^ Discs are the largest immune‐privileged, low‐nutrient, avascular organs in the body.^24^ Compared with peripheral disc AF cells, central disc NP cells depend on diffusion from blood vessels at the disc margins to obtain nutrients.^25^ Therefore, decreased blood supplies, subchondral bone sclerosis, and endplate calcification, which occur during mechanical stress, injury, smoking, and aging, can reduce the transport of nutrients to discs and is suspected to contribute to disc degeneration.^25^

In humans, intervertebral disc degeneration begins during early childhood,^26^, ^27^ and is generally more severe in the NP than in the AF.^27^ In the NP, obvious clefts and radial tears can occur between ages 11‐16 years.^27^ Aggrecan biosynthesis and type II procollagen content peak at ages ≤5 years and diminish between ages 5 and 15 years, whereas the percentage of denatured type II collagen increases after 5 years of age.^26^ Approximately 40% of people under 30 years of age and 90% of those over 55 years of age present with lumbar disc degeneration.^28^ A reduction in cell numbers, another major characteristic of disc degeneration, primarily results from programmed cell death, or apoptosis.^29^ Apoptotic cells increase substantially between the ages of 11 and 16 years, associated with the disappearance of notochordal cells and chondrocyte proliferation,^27^ suggesting a potential link between apoptosis and notochordal cell disappearance during the pathogenesis of disc degeneration.^8^ Furthermore, a notably high incidence of apoptosis has been observed in human aged and degenerated discs^30^ and rodent degenerative discs by static compression.^23^, ^31^ The incidence of irreversible cell growth arrest due to aging, or senescence,^32^ also increases during human disc degeneration.^33^, ^34^ Understanding of this unique, harsh, low‐glucose, low‐oxygen, low‐pH and high‐osmolality environment and the load fluctuations that disc cells are exposed to is essential for designing new biological therapies to prevent disc aging and degeneration.

### 1.3 Autophagy and mTOR signaling

**Figure 1:**
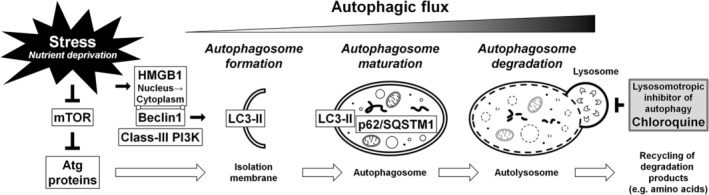
Schematic illustration of disc cellular autophagy. Under stress conditions, for example, nutrient deprivation, the mammalian target of rapamycin (mTOR), a signal integrator that detects nutrients to signal the execution of cell growth and division, is suppressed, which initiates autophagy through the activation of autophagy‐related (Atg) genes and proteins. High‐mobility group box 1 (HMGB1), which is involved in stress response, translocates from the nucleus to the cytoplasm and directly interacts with Beclin1 (Atg6 homolog). The Beclin1–class‐III phosphatidylinositol 3‐kinase (PI3K) complex initiates autophagosome formation by developing the isolation membrane. Autophagosome maturation is completed by the growth and closure of the isolation membrane, driven by the conjugation of phosphatidylethanolamine with light chain 3 (LC3) (Atg8 homolog), leading to the formation of the autophagosome‐membrane‐bound form LC3‐II. Then, p62/sequestosome 1 (p62/SQSTM1) and p62/SQSTM1‐bound polyubiquitinated proteins become incorporated into completed autophagosomes. The completed autophagosome fuses with the lysosome to form the autolysosome (which can be inhibited by chloroquine), where the enclosed cargo is degraded, and its constituents are released and recycled. Understanding of autophagy requires monitoring this dynamic, multi‐step process of autophagic flux. In our previous time‐course observational study, the graded supply of serum and nutrients decreased proliferation and metabolic activity and increased autophagy, apoptosis, and senescence in rabbit disc annulus fibrosus cells

Autophagy, the intracellular process during which cells degrade and recycle damaged components, is an important cell survival mechanism that sustains metabolism and prevents the accumulation of damaged, toxic proteins and organelles under stress conditions, especially nutrient deprivation.^35^, ^36^, ^37^, ^38^ Autophagy involves autophagy‐related (Atg) genes and proteins.^36^, ^37^ Under typical physiological conditions, Atg proteins are expressed at relatively low levels; however, basal autophagy serves as the quality‐control machinery for cellular renovation and homeostasis.^35^, ^38^ Under stress conditions, the activation of Atg proteins results in the formation and maturation of the autophagosome, which captures damaged organelles, misfolded proteins, and invading microorganisms in induced autophagy.^35^, ^36^, ^37^ The completed autophagosome then fuses with the lysosome, to form the autolysosome, which degrades the enclosed cargo and releases its constituents for reuse.^35^, ^36^, ^37^ The microtubule‐associated protein 1 light chain 3 (LC3) (a mammalian homolog of yeast Atg8) is a ubiquitin‐like protein with cytosolic (LC3‐I) and phosphatidylethanolamine‐conjugated (LC3‐II) forms.^39^, ^40^ LC3‐II is the only protein that remains attached to the autophagosome membrane after formation, making it in a robust marker for ongoing autophagy.^39^, ^40^ Measuring LC3‐II protein expression by Western blotting and/or counting LC3 puncta during immunofluorescence can be used to determine autophagic activity, as increased LC3‐II expression and LC3 puncta indicate an increased number of autophagosomes.^40^ High‐mobility group box 1 (HMGB1) is a nuclear DNA‐binding protein and an extracellular damage‐associated molecular pattern molecule.^41^ In response to stress, HMGB1 translocates from the nucleus to the cytoplasm, before being released extracellularly.^41^ Cytoplasmic HMGB1 directly interacts with Beclin1 (Atg6 homolog), resulting in autophagosome formation.^40^, ^41^ p62/sequestosome 1 (p62/SQSTM1) is a ubiquitin‐binding protein that acts as a link between LC3 and ubiquitinated substrates.^40^ p62/SQSTM1 and p62/SQSTM1‐bound polyubiquitinated proteins become incorporated into the completed autophagosome and are degraded in the autolysosome; therefore, their expression levels inversely correlate with autophagosome degradation levels.^40^ Monitoring this dynamic, sequential process, known as autophagic flux, is essential to understand the roles played by autophagy (Figure 1).

**Figure 2:**
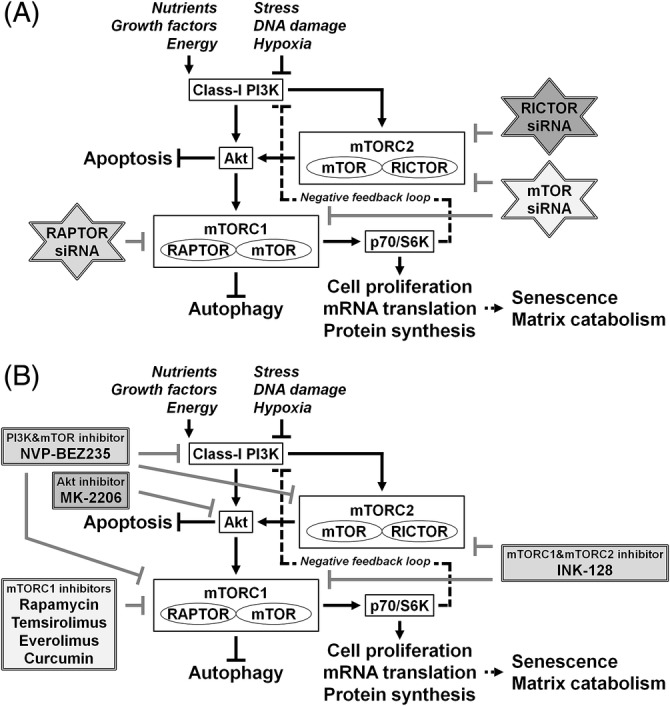
Schematic illustration of the RNA interference (RNAi)‐mediated and pharmacological modulation of disc cellular mTOR signaling. The mammalian target of rapamycin (mTOR) is a serine/threonine kinase that detects nutrients to signal the execution of cell growth and division. mTOR exists in two complexes: mTOR complex 1 (mTORC1), which contains regulatory‐associated protein of mTOR (RAPTOR), and mTOR complex 2 (mTORC2), which contains rapamycin‐insensitive companion of mTOR (RICTOR). mTORC1 acts as a signal integrator for nutrients, growth factors, energy, stress, DNA damage, and hypoxia. Down‐stream effectors of mTORC1, including p70/ribosomal S6 kinase (p70/S6K), regulate cell proliferation, messenger RNA (mRNA) translation, and protein synthesis. Autophagy, an intracellular degradation system, is under the tight negative regulation of mTORC1. mTORC1 can be regulated upstream by Akt, an essential prosurvival mediator that suppresses apoptotic cell death. Akt phosphorylation is governed by the class‐I phosphatidylinositol 3‐kinase (PI3K) and mTORC2. Furthermore, a negative feedback loop exists between p70/S6K and the class‐I PI3K. (A) In our previous study to modulate mTOR signaling by RNAi, small interfering RNAs (siRNAs) against mTOR, RAPTOR, and RICTOR were applied. In human disc nucleus pulposus cells, RNAi treatments clarified mTOR‐dependent senescent cell aging and extracellular matrix catabolism. The selective suppression of mTORC1/RAPTOR, but not the extensive suppression of mTORC1/mTORC2/mTOR and mTORC2/RICTOR, protected against inflammation‐induced disc cellular apoptosis, senescence, and matrix catabolism through the induction of autophagy and Akt. (B) In our previous study to pharmacologically modulate mTOR signaling, mTORC1 inhibitors, such as rapamycin, temsirolimus, everolimus, and curcumin, a dual mTORC1 and mTORC2 inhibitor, INK‐128, a dual PI3K and mTOR inhibitor, NVP‐BEZ235, and/or an allosteric Akt inhibitor, MK‐2206, were examined. In human disc nucleus pulposus cells, protective effects were observed for mTORC1, but not mTORC2, inhibitors against inflammation‐induced disc cellular apoptosis, senescence, and matrix catabolism through the induction of autophagy and Akt

Autophagy is negatively regulated by the mammalian target of rapamycin (mTOR).^42^ mTOR is a serine/threonine kinase that integrates nutrients, growth factors, energy, and stress signals to trigger the activation cell growth and division.^42^ mTOR exists in two complexes: mTOR complex 1 (mTORC1), which contains regulatory‐associated protein of mTOR (RAPTOR), and mTOR complex 2 (mTORC2), which contains rapamycin‐insensitive companion of mTOR (RICTOR).^42^ Downstream mTORC1 effectors, including p70/ribosomal S6 kinase (p70/S6K), regulate cell proliferation, messenger RNA (mRNA) translation, and protein synthesis.^42^ mTORC1 is regulated upstream by Akt, an essential prosurvival mediator that suppresses apoptosis.^43^ Moreover, Akt phosphorylation has been associated with class‐I phosphatidylinositol 3‐kinase (PI3K) and mTORC2.^42^, ^43^ Because mTOR is the central signal integrator for nutrition,^42^ the extensive modulation of mTOR signaling would be harmful, and homozygous mTOR deletion results in embryonic lethality.^44^ Therefore, identifying which subunit(s) of mTOR exert effects on target cells is necessary (Figure 2A,B).

### 1.4 Autophagy and mTOR signaling in the intervertebral disc

Autophagy has previously been studied in articular chondrocytes. In the mouse knee cartilage, spontaneous aging and experimental osteoarthritis induced by the progressive transection of the medial meniscotibial and collateral ligaments both reduced Atg protein expression.^45^ Traumatic impaction injury also suppressed autophagy.^46^ Mechanical damages have been partially rescued by the administration of rapamycin, an autophagy inducer through mTORC1 inhibition.^46^, ^47^ In human chondrocytes, protective effects of rapamycin against inflammation‐induced apoptosis and catabolic gene expression have been shown, in vitro.^48^ Compared with femoral head cartilage derived from surgically treated femoral neck fracture patients (nondegenerated), LC3 expression increased in lateral femoral condyle cells (mildly degenerated) and decreased in medial femoral condyle cells (severely degenerated) derived from patients with varus‐type knee osteoarthritis, in vivo.^48^ Osteoarthritis severity‐dependent decreases in Atg protein expression have been observed during a different human articular cartilage study.^45^ These may be caused by the stress‐response induction of autophagy.^36^, ^37^ Chondrocytes may induce autophagy in response to stress, such as inflammation and injury, during earlier osteoarthritis stages. However, cells are thought to lose the reaction potential of autophagy during advanced osteoarthritis, requiring arthroplasty.

Compared with osteoarthritis, the involvement of autophagy during degenerative disc disease has not been fully clarified. In human^49^ and rat discs,^50^, ^51^ autophagy activation has been confirmed by electron microscopy. In rat disc NP tissues, Atg protein expression increased with spontaneous aging.^50^ In chemically induced type‐I diabetic rat discs, the accelerated development of mild degeneration, associated with stress‐response autophagy, apoptosis, and senescence induction, has been observed.^51^ Differential autophagy levels between disc and cartilage tissues may be caused by the presence of NP notochordal cells in rodents.^8^, ^9^ Because of their markedly slow proliferation and prolonged maintenance,^52^ notochordal cells may require autophagy induction with aging and in response to disease stress. Furthermore, the upregulation of *Atg* gene expression was observed in human discs with the Pfirrmann^53^ grades 4 to 5, compared with grades 1 to 3^49^; however, the confirmation of autophagic flux at either the protein or organelle levels, using LC3‐I to LC3‐II conversion and p62/SQSTM1 degradation, was not performed in these cells. Disc cellular autophagy has gained increasing interest, including the activation in both disc NP and AF cells in response to a variety of stress conditions, such as nutrient deprivation,^54^, ^55^, ^56^, ^57^ oxidative stress,^58^, ^59^, ^60^ compression overload,^61^ inflammation,^62^ hyperlactatemia,^63^ hyperosmolarity,^64^ and hypoxia.^65^ Meanwhile, evidence regarding mTOR signaling in disc cells is still limited.^64^, ^65^, ^66^ Therefore, mechanistic investigations to clarify the roles played by autophagy and mTOR signaling must be conducted. Furthermore, the clinical relevance of autophagy and mTOR signaling during degenerative disc disease remains largely unexplored.

### 1.5 Hypothesis

We hypothesized that resident cells would utilize autophagy and mTOR signaling to cope with low‐nutrient, stressful conditions of the disc.^25^ However, little evidence exists regarding the effects of autophagy and mTOR signaling on disc cell and tissue homeostasis. Therefore, we performed in vitro and in vivo studies of human and rabbit disc cells and tissues, modulated by the graded supply of nutrition, RNA interference (RNAi), and pharmacological agents, to elucidate the involvement and roles played by autophagy and mTOR signaling in the intervertebral disc. This brief review introduces our study findings and interpretations, discussing the future applications of therapeutically modulating biological autophagy and/or mTOR signaling to treat degenerative disc disease.

## 2 SERUM AND NUTRIENT DEPRIVATION IN RABBIT DISC CELLS

Published studies have not confirmed or mechanistically examined autophagic flux in intervertebral disc cells. Furthermore, many in vitro studies have been performed using conventional cell‐culture conditions, such as 10% to 20% serum supplementation and normoxia, which differ considerably from the in vivo disc environment.^25^ Therefore, as a preliminary study, we carefully and systematically examined intervertebral disc cell fate, in vitro.^67^ We conducted time‐course experiments to measure autophagic flux in rabbit disc AF cells under varying degrees of nutrient withdrawal by applying media with various serum concentrations. In this study, a conventional monolayer cell culture system was used due to the difficulty observing detailed changes in intracellular signaling networks and autophagic flux using 3D cell culture systems. Although in vitro monolayer cultures may limit physiological relevance to in vivo situations,^68^ the laminar AF is better simulated by monolayers than the gelatinous NP. All experiments were performed in 5% O_2_ to simulate the physiological environment of disc AF cells, as the concentration of oxygen in the bone marrow, whose vasculature supplies discs, is approximately 4% to 7%.^69^ We used rabbit cells due to reduced phenotypic variability, including age, sex, and degeneration grade, compared with surgically obtained human cells. Hence, the objective of our first study was to clarify the fundamental relationships between nutrient supply and autophagy, apoptosis, and senescence levels in rabbit disc AF cells (Figure 1).^67^

### 2.1 Decreased disc cell proliferation and metabolic activity under reduced serum and nutrient conditions

To characterize disc cellular responses to changes in serum‐related nutrient supply, we investigated cell proliferation in Hank's balanced salt solution (HBSS) or Dulbecco's modified Eagle's medium (DMEM), containing 0% to 10% fetal bovine serum (FBS). Cell numbers decreased in HBSS and 0% FBS‐supplemented DMEM, remained unchanged in 1% FBS‐supplemented DMEM, and increased in 10% FBS‐supplemented DMEM. Although serum deprivation reduced cell numbers, the nutrients supplied by DMEM containing 1% FBS were sufficient to maintain AF disc cell numbers.

To evaluate disc cell metabolism, we measured cell dehydrogenase activity and DNA amount in HBSS or DMEM, containing 0% to 20% FBS. Total dehydrogenase activity and DNA amount both decreased under limited‐serum and limited‐nutrient conditions. Normalized cell metabolic activity (dehydrogenase activity/DNA amount) remained relatively unchanged in DMEM containing 3% to 20% FBS but decreased in DMEM containing 0% to 1% FBS. Normalized cell metabolic activity showed dropped sharply in HBSS. Disc AF cells responded to nutrient deprivation by reducing cell proliferation and metabolism. Further investigations aim to identify the optimal culture conditions for rabbit disc AF cells in 5% O_2_, although DMEM containing 5% FBS resulted in the highest normalized cell metabolic activity.^67^

### 2.2 Increased disc cellular autophagy under reduced serum and nutrient conditions

To monitor autophagic flux in disc AF cells, we performed time‐course LC3 puncta counting and nuclear and cytoplasmic HMGB1 measurements, using imaging cytometry. Although the number of LC3 puncta per cell peaked after 12 hours in DMEM containing 1% to 10% FBS, more pronounced increases in LC3 puncta were observed in HBSS and 0% FBS‐supplemented DMEM, peaking at 24 to 48 hours. Nuclear HMGB1 intensity peaked at 12 to 24 hours, whereas cytoplasmic HMGB1 intensity increased over time, in HBSS and DMEM containing 0% to 1% FBS. Thus, the degree of LC3 puncta and cytoplasmic HMGB1 changes corresponded to the severity of nutrient withdrawal. Reduced serum and nutrient conditions activate the early (cytoplasmic HMGB1 translocation) and middle (LC3 puncta formation) phases of disc cellular autophagic flux (Figure 1).

We also performed Western blotting for LC3, HMGB1, and a selective autophagy substrate, p62/SQSTM1. Western blotting results demonstrated time‐dependent increases in LC3‐II in 0% FBS‐supplemented DMEM, transient increases at 12 hours in 1% FBS‐supplemented DMEM, and no obvious increases in 10% FBS‐supplemented DMEM. Likewise, total (nuclear + cytoplasmic) HMGB1 increased markedly at 12 hours and time‐dependent p62/SQSTM1 decreases were observed in DMEM containing 0% FBS. Reduced serum concentrations further activated the late phase (p62/SQSTM1 degradation) of disc cellular autophagic flux (Figure 1).

An autophagy inhibitor, chloroquine, used to induce the autophagosome (LC3‐II) accumulation by blocking lysosomal acidification,^40^ increased LC3‐II levels for all serum concentrations, even nutrient‐rich 10% FBS, indicating the presence of basal physiological autophagy. Furthermore, differences in LC3‐II levels with and without chloroquine were consistent with the severity of FBS withdrawal, indicating the induction of stress‐response autophagy during nutrient deprivation in disc AF cells (Figure 1).

### 2.3 Increased disc cellular apoptosis, senescence, and autophagy under reduced serum conditions

To examine other mechanisms that occur in disc AF cells under nutrient deprivation conditions, we measured apoptosis and senescence. In staining assays, the percentage of apoptotic terminal deoxynucleotidyl transferase dUTP nick end labeling (TUNEL)^70^ ‐positive cells and nuclear cleaved caspase‐3^71^ ‐positive cells increased with decreasing FBS concentrations. The percentage of senescence‐associated β‐galactosidase (SA‐β‐gal)^72^ ‐positive cells and nuclear p16/INK4A^73^ ‐positive cells also increased with diminishing FBS concentrations.

Western blotting also showed increased LC3‐II, HMGB1, cleaved caspase‐3, and p16/INK4A levels and decreased p62/SQSTM1 levels with FBS withdrawal, indicating the concurrent induction of disc cellular autophagy, apoptosis, and senescence under limited nutrition conditions.

Subsequently, we examined the co‐localization of autophagy and senescence markers in cells positive for an apoptotic marker by multi‐color immunofluorescence. After the withdrawal of FBS, “apoptotic” nuclear cleaved caspase‐3‐positive cells presented a higher percentage of immunopositivity for “autophagic” cytoplasmic LC3 than for “senescent” nuclear p16/INK4A. “Non‐apoptotic” but “senescent” cells negative for nuclear cleaved caspase‐3 but positive for p16/INK4A also showed “autophagic” accumulation of LC3 puncta; however, the degree of LC3 accumulation was markedly lower than in “apoptotic” cells positive for nuclear cleaved caspase‐3. These disc AF‐cell findings indicate the involvement of autophagy during both apoptosis and senescence. Then, the degree of autophagic response in apoptosis might be greater than in senescence. However, the observed severity of autophagic response would depend on conditions of cell passage number, nutrient abundance, oxygen concentration, and treatment strength and duration. Further studies for intervertebral disc cell fate are warranted.

### 2.4 Cytoplasmic HMGB1 as an autophagy marker

Compared with well‐established LC3‐II and p62/SQSTM1 to monitor autophagy,^40^ time‐dependent changes in the expression and localization of HMGB1 observed by independent nuclear and cytoplasmic HMGB1 measurements is noteworthy, suggesting the usefulness of HMGB1 as an autophagy sensor in adult disc AF cells, similar to embryonic fibroblasts and cancer cells.^41^ Western blotting for total (nuclear + cytoplasmic) HMGB1 expression supported stress‐reactive cytoplasmic translocation, followed by extracellular release, of HMGB1 nuclear protein. However, in this study, nuclear HMGB1 levels changed under limited nutrition unlike embryonic and cancer cells.^41^ Therefore, we examined the extent to which markers of autophagy were concurrently present in apoptotic cells.

Multicolor immunofluorescence detected that many “apoptotic” nuclear cleaved caspase‐3‐positive cells simultaneously showed marked nuclear HMGB1 elevation as well as LC3 puncta formation and cytoplasmic HMGB1 release. Nuclear retention of HMGB1 occurs during apoptosis because of posttranslational modifications that affect chromatin binding.^74^ Thus, roles of HMGB1 in disc disease need to be further studied. However, these findings are the first to support cytoplasmic HMGB1 as an autophagy marker in disc AF cells.

### 2.5 Summary

Serum and nutrient deprivation decreased rabbit disc AF cell proliferation and metabolic activity and increased autophagy, apoptosis, and senescence.^67^ Serum deprivation‐induced autophagy in disc cells is consistent with prior reports.^54^, ^55^, ^56^, ^57^ Our findings support the understanding of disc cell fate and the monitoring of autophagic flux. The prosurvival effects of autophagy are well‐known among diverse cell types,^75^ including disc cells^57^, ^64^, ^76^ and chondrocytes.^46^, ^47^, ^48^ However, further mechanistic investigations of the intersection between autophagy, apoptosis, and senescence under clinically relevant conditions remain necessary.

## 3 RNAI‐MEDIATED MTOR SIGNALING MODULATION IN HUMAN DISC CELLS

The substantial involvement of autophagy in disc cells suggests the potential for autophagy‐modulating therapies during degenerative disc disease. Autophagy is negatively controlled by intracellular mTOR signaling,^42^ and many autophagy‐inducing and autophagy‐inhibiting agents exert their effects through mTOR signaling.^40^ However, due to cross‐talk and redundancy in the mTOR‐signaling network,^42^, ^77^ the specific pharmacological induction and inhibition of mTOR signaling remain difficult. The chronic administration of rapamycin, a selective mTORC1 inhibitor and autophagy inducer, can inhibit mTORC2.^78^ A PI3K inhibitor, 3‐methyladenine, effectively blocks the early phase of autophagy, through class‐III PI3K inhibition‐mediated Beclin1 inactivation,^40^ and also inhibits class‐I PI3K, promoting autophagy even at suboptimal concentrations during long‐term experiments.^79^ Therefore, we utilized RNAi to knockdown specific mTOR‐signaling cascades,^80^ using small interfering RNAs (siRNAs) against mTOR, RAPTOR, and RICTOR. To exclude off‐target effects of RNAi,^81^ findings were confirmed using at least two siRNAs, with different sequences, for all assays.

In this study, to assess clinical relevance, we utilized human disc NP cells obtained from patients who underwent lumbar interbody fusion surgeries to treat degenerative disease. To simulate the physiologically hypoxic disc NP environment,^25^ experiments were performed in 2% O_2_. Understanding of disc NP‐cell fate and intracellular signaling is essential as the pathology of degenerative disc disease is widely thought to be originated from NP.^27^ Moreover, compared with disc AF cells, NP cells are susceptible against the environmental changes in nutrient deprivation, hypoxia, and inflammation,^82^ thereby suggesting a deeper involvement of autophagy and mTOR signaling. The inclusion of two different cell types, NP and AF, would further strength the experimental results in this review.

In addition, interleukin‐1 beta (IL‐1β) was used to simulate a clinically relevant inflammatory disease condition.^83^ IL‐1β is a proinflammatory cytokine closely linked to the pathogenesis^83^ and severity of disc degeneration.^84^ In fact, there are complex cross‐talk mechanisms between mTOR signaling and inflammation, for example, IL‐1β and tumor necrosis factor‐alpha.^85^ Akt promotes nuclear factor kappa‐light‐chain‐enhancer of activated B cells (NF‐κB) inhibitor kinase‐dependent regulation of NF‐κB signaling through mTORC1.^86^, ^87^ In our observation, IL‐1β administration stimulated mTORC1 and p70/S6K but also induced autophagy presented with increased LC3‐II and decreased p62/SQSTM1 in human disc NP cells.^80^ Effects of inflammation on disc cellular autophagy and mTOR signaling are subjects to be studied in the future.

The objective of our second study was to elucidate the involvement and roles played by mTOR signaling in human disc NP cells (Figure 2A).^80^

### 3.1 Consistent disc cellular autophagy and differential Akt activation by the selective interference of mTOR signaling

First, we performed Western blotting on disc cell protein extracts, confirming specific decreases in the expression levels of mTOR, RAPTOR, and RICTOR proteins caused by the corresponding siRNAs. Western blotting further showed that mTOR and RICTOR siRNAs decreased downstream p70/S6K and upstream Akt phosphorylation, whereas RAPTOR siRNA decreased p70/S6K phosphorylation but increased Akt phosphorylation. Akt activation occurs through the negative feedback loop between p70/S6K and class‐I PI3K.^88^, ^89^, ^90^ Class‐I PI3K is responsible for various cellular functions related to the activation of Akt.^42^ Therefore, the suppression of mTORC1 and p70/S6K by RAPTOR RNAi resulted in the loss of class‐I PI3K feedback and subsequent Akt activation.^89^ In greater details, at baseline, the mTORC1/p70/S6K axis suppresses the upstream PI3K/Akt signaling pathway through the phosphorylation of insulin receptor substrate 1 (IRS1),^90^, ^91^ a second messenger from insulin‐like growth factor 1 receptor to PI3K.^88^ Then, mTORC1 inhibition‐mediated relief of this negative feedback loop activates IRS1, PI3K, Akt, and ERK in some cell types.^92^, ^93^ This feedback system has been found in a variety of cancers^90^, ^91^, ^92^, ^93^, ^94^, ^95^, ^96^, ^97^ and also non‐malignant intervertebral disc cells (Figure 2A).^80^, ^98^

Western blotting results demonstrated that all three siRNAs increased LC3‐II and decreased p62/SQSTM1 levels, which are consistent with enhanced autophagy induction,^40^ indicating successful RNAi‐mediated mTORC1 suppression (Figure 2A).^42^

### 3.2 Differential effects on disc cell proliferation, viability, apoptosis, senescence, and matrix metabolism by the selective interference of mTOR signaling

To examine disc cellular responses to RNAi, we measured cell proliferation and dehydrogenase activity. Cell numbers decreased in response to mTOR and RICTOR siRNAs, but not RAPTOR siRNA. Total dehydrogenase‐based cell viability also decreased following mTOR and RICTOR siRNAs, but not RAPTOR siRNA. Cell cycle and proliferation are positively controlled by translation‐stimulating p70/S6K, which is positively regulated by mTORC1 and negatively regulated by translation‐repressing eukaryotic translation initiation factor 4E‐binding protein 1 (4E‐BP1), which is, in turn, negatively regulated by mTORC1.^42^ Therefore, in this study, all RNAi treatments should facilitate the negative regulation of disc cell cycle and proliferation through mTORC1 suppression. However, the relatively unchanged disc cell proliferation and viability following mTORC1/RAPTOR RNAi may be due to differences in Akt phosphorylation levels. Akt increases cell proliferation through mTORC1 induction (p70/S6K induction and 4E‐BP1 inhibition) and other pathways, including the inhibition of negative cell‐cycle regulators p27/KIP1 and p21/CIP1.^43^ Thus, increased Akt phosphorylation after mTORC1/RAPTOR RNAi, associated with the negative feedback loop between p70/S6K and class‐I PI3K,^88^, ^89^, ^90^ resulted in differential effects on disc cell proliferation and viability from mTORC1/mTORC2/mTOR and mTORC2/RICTOR RNAi.

We then assessed RNAi effects on disc cell death, aging, and matrix metabolism. TUNEL staining showed that proinflammatory cytokine IL‐1β‐induced apoptotic cells decreased after RAPTOR siRNA only. Western blotting showed that IL‐1β‐induced apoptotic markers were decreased by RAPTOR siRNA only. Differential anti‐apoptotic effects associated with mTORC1/RAPTOR RNAi, compared with mTORC1/mTORC2/mTOR and mTORC2/RICTOR RNAi, may also be due to increased Akt phosphorylation. A major role of Akt is to enhance cell survival, by blocking proapoptotic proteins, for example, Bcl‐2‐associated death promoter protein, and through effects on transcription factors, for example, forkhead box O and p53.^43^ Collectively, Akt‐mediated survival and proliferation strongly support mTORC1/RAPTOR interference as a potential therapeutic application for intervertebral disc disease.

Western blotting results showed that IL‐1β‐induced senescent markers were decreased by all mTOR, RAPTOR, and RICTOR siRNAs. Similar findings were observed during SA‐β‐gal staining. These consistent anti‐senescent RNAi findings are likely caused by mTORC1 suppression, resulting in p70/S6K inhibition and 4E‐BP1 induction. 4E‐BP1 is required for dietary restriction‐induced lifespan extensions,^99^ and the deletion of p70/S6K also extends lifespans.^100^ mTOR‐hypomorphic mice increase lifespans with reduced senescent p16/INK4A expression.^101^ Hence, our findings of cellular senescence suggested the disruption of mTORC1‐mediated proliferation. In this review, we try to provide a possible interpretation that mTORC1/RAPTOR interference vs mTORC1/mTORC2/mTOR and mTORC2/RICTOR interference exerts different responses in apoptosis and similar responses in senescence under proinflammatory IL‐1β stimulation; however, the complexity between disc cell fate and intracellular signaling warrants further mechanistic clarification.

Western blotting showed that the IL‐1β‐induced release of catabolic MMP‐3 and MMP‐13 into cultured supernatants was markedly decreased by mTOR, RAPTOR, and RICTOR siRNAs. Gelatin zymography also showed that the IL‐1β‐induced activation of MMP‐2 and MMP‐9 were effectively decreased by RAPTOR siRNA. Real‐time reverse transcription‐polymerase chain reaction (RT‐PCR) further demonstrated the IL‐1β‐induced upregulation of catabolic *MMP‐3*, *MMP‐13*, and *ADAMTS‐4* genes was suppressed by RAPTOR siRNA. IL‐1β‐induced the downregulation of anabolic *ACAN*, encoding aggrecan, and *COL2A1*, encoding collagen type II alpha 1 chain, was not completely rescued by RAPTOR siRNA; however, RAPTOR siRNA treatment resulted in a trend toward the upregulation of these anabolic genes. Only a few reports have described the regulation and mechanisms underlying matrix metabolism by mTOR. Articular cartilage‐specific mTOR deletions have been shown to be protective against destabilized medial meniscus‐induced osteoarthritis in mice, based on increased autophagy, decreased apoptosis, and decreased catabolic MMP‐13 expression.^102^ Our anti‐catabolic RNAi findings suggested that the expression and activation of MMPs potentially depend on mTORC1‐mediated mRNA translation and protein synthesis, although the possibility of other regulatory mechanisms including RNA transcription and mRNA stability cannot be excluded. Further investigations are required; however, mTORC1‐mediated proliferation and translation provide a plausible interpretation for reduced disc cell aging and matrix catabolism in response to mTOR, RAPTOR, and RICTOR RNAi treatments.

### 3.3 Summary

This is the first mechanistic study to demonstrate cascade‐dependent, differential roles for mTOR signaling in human disc NP cells.^80^ Although the extensive RNAi‐mediated suppression of mTORC1/mTORC2/mTOR and mTORC2/RICTOR was not always advantageous to disc cells, the selective suppression of mTORC1/RAPTOR protected against inflammation‐induced disc cellular apoptosis, senescence, and matrix catabolism.^80^ The beneficial effects of mTORC1/RAPTOR interference were dependent on the activation of autophagy and Akt.^80^

## 4 PHARMACOLOGICAL MTOR SIGNALING MODULATION IN HUMAN DISC CELLS

Although the RNAi‐mediated modulation of mTOR signaling identified protective effects for mTORC1/RAPTOR suppression in human disc NP cells,^80^ pharmacological modulation is favorable compared with gene‐silencing therapy in clinical practice due to safety issues. Rapamycin, an mTORC1 inhibitor, extends mammalian lifespan^103^ and plays protective roles in chondrocytes.^46^, ^47^, ^48^ Despite the effectiveness of rapamycin,^46^, ^47^, ^48^, ^103^ rapamycin is not widely used clinically due to poor water solubility, which limits administration routes, and serious adverse effects, including immunosuppression.^104^ Rapamycin derivatives, such as everolimus and temsirolimus, have been developed.^105^ Everolimus is only available orally,^105^ whereas temsirolimus is a prodrug that can be administered both intravenously and orally.^105^ Including rapamycin, these agents represent first‐generation mTOR inhibitors.^105^ A natural polyphenol from the rhizomes of turmeric, curcumin, has also been reported to inhibit mTOR signaling in various cancer cells.^106^ Therefore, we performed an additional in vitro study to identify the most suitable mTORC1 inhibitor for human degenerative disc disease treatment.^98^ We also tested more recently developed second‐generation mTOR inhibitors: a dual mTORC1 and mTORC2 inhibitor, INK‐128, and a dual PI3K and mTOR inhibitor, NVP‐BEZ235.^105^ Both are in clinical trials for advanced solid tumor treatment.^105^ To clarify the role of increased Akt phosphorylation during mTORC1/RAPTOR interference in human disc NP cells, an allosteric Akt inhibitor, MK‐2206, was tested, which is also in clinical trials for renal cell carcinoma (Figure 2B).^107^

### 4.1 Consistent disc cellular autophagy induction and Akt activation by pharmacological mTORC1 inhibition

To identify drug toxicity, we assessed dose‐dependent disc cell viability and determined that a 100 nM dose was effective but non‐toxic for rapamycin, temsirolimus, everolimus, and curcumin.

Western blotting demonstrated that mTORC1‐inhibiting rapamycin, temsirolimus, and everolimus successfully decreased mTOR and downstream p70/S6K phosphorylation but increased upstream Akt phosphorylation; however, curcumin did not affect phosphorylation. These mTORC1 inhibition‐induced changes in mTOR and related feedback signaling molecules^88^, ^89^, ^90^ were consistent with the mTORC1/RAPTOR RNAi results (Figure 2B).^80^

Western blotting results showed that rapamycin, temsirolimus, and everolimus increased LC3‐II and decreased p62/SQSTM1 levels, indicating enhanced autophagy activation.^40^ Additional Western blotting following chloroquine treatments^40^ revealed that autophagic LC3‐II accumulation was most prominent in temsirolimus‐treated cells (Figure 2B).

### 4.2 Protective effects of pharmacological mTORC1 inhibition against disc cellular apoptosis, senescence, and matrix catabolism

Western blotting, TUNEL staining, and SA‐β‐gal staining showed that IL‐1β‐induced apoptosis and senescence were suppressed by mTORC1 inhibitors, which was consistent with the mTORC1/RAPTOR RNAi results.^80^ This trend was distinct for rapamycin, temsirolimus, and everolimus treatments compared with curcumin treatments.

Western blotting in cultured supernatants showed that the IL‐1β‐induced release of catabolic MMP‐2, MMP‐3, MMP‐9, and MMP‐13 was decreased by rapamycin, temsirolimus, and everolimus. Real‐time RT‐PCR further demonstrated that IL‐1β‐induced downregulation of *ACAN* and *COL2A1* expression was reversed by first‐generation mTOR inhibitors. The pharmacological mTORC1 inhibition results were all similar to those for RNAi‐mediated suppression of mTORC1/RAPTOR.^80^

The tested mTORC1 inhibitors induced anti‐apoptosis, anti‐senescence, and anti‐matrix catabolism in disc NP cells. Rapamycin, temsirolimus, and everolimus showed similar mTORC1 inhibition effects; however, temsirolimus appeared to be the most effective, possibly as a result of improved water solubility, which also enables intravenous administration, whereas the other inhibitors are limited to oral administration.^105^, ^106^ In contrast, curcumin was not toxic but was less effective, and poor aqueous solubility, low bioavailability, and intense color‐staining are limitations associated with curcumin.^108^

### 4.3 Nonprotective effects of pharmacological dual mTOR inhibition against disc cellular apoptosis, senescence, and matrix catabolism

We additionally tested second‐generation mTOR inhibitors, INK‐128 and NVP‐BEZ235, which block multiple mTOR‐signaling molecules. Based on cell viability results, 100 nM was used to compare effects at the same dose as the first‐generation mTORC1 inhibitors.

Western blotting showed that INK‐128 and NVP‐BEZ235 both decreased mTOR, p70/S6K, and Akt phosphorylation, indicating successful mTOR inhibition. However, unlike mTORC1 inhibitors, dual mTOR inhibitors did not induce autophagy, despite mTORC1 inhibition (Figure 2B).

Furthermore, INK‐128 and NVP‐BEZ235 did not suppress IL‐1β‐induced apoptotic and senescent effects. In contrast, both agents accelerated apoptosis and senescence. Western blotting showed that INK‐128 and NVP‐BEZ235 promoted IL‐1β‐induced catabolic MMP‐2, MMP‐3, MMP‐9, and MMP‐13 release to cultured supernatants. Thus, second‐generation mTOR inhibitors did not protect human disc NP cells against inflammation, consistent with the results of mTORC1/mTORC2/mTOR and mTORC2/RICTOR RNAi.^80^

### 4.4 Akt‐dependent effects of pharmacological mTORC1 inhibition on disc cellular proautophagy, anti‐apoptosis, anti‐senescence, and anti‐matrix catabolism

We examined the Akt inhibitor MK‐2206 at 5 μM, based on cell viability assay results and prior reports.^109^

Western blotting showed that MK‐2206, either with or without temsirolimus, decreased mTOR, p70/S6K, and Akt phosphorylation, indicating successful Akt inhibition. Temsirolimus did but MK‐2206 did not enhance autophagy. Despite the negative regulation of autophagy by mTORC1 followed by Akt,^42^ dual mTOR inhibitors and MK‐2206 with extensive suppression of PI3K/Akt/mTOR signaling did not activate autophagy in human disc cells. Similar responses are observed in human lymphoblasts, in which accelerated autophagy at early time points but switched autophagy to apoptosis are observed by MK‐2206 supplementation.^110^ Further studies are required to disclose the presence of other cross talks through PI3K, mTORC2, and Akt to mTORC1 and autophagy (Figure 2B).

Under IL‐1β stimulation, apoptosis and senescence were suppressed by temsirolimus but exacerbated by MK‐2206. Similarly, IL‐1β‐induced catabolic MMP release was suppressed by temsirolimus but stimulated by MK‐2206. Based on the proapoptotic, prosenescent, and procatabolic findings associated with the Akt inhibition by MK‐2206, the protective effects of temsirolimus against inflammation primarily depend on Akt activation in human disc NP cells. Akt1 regulates osteophyte formation in osteoarthritis as well as endochondral ossification during skeletal growth in mice.^111^ Accordingly, Akt is the key regulator of mTOR signaling in human disc cells.

### 4.5 Summary

In human disc NP cells, mTORC1 inhibitors, but not dual mTOR inhibitors, protected against inflammation‐induced apoptosis, senescence, and matrix catabolism.^98^ The beneficial effects of mTORC1 inhibitors depended on the induction of autophagy and Akt.^98^ Based on these results, we propose temsirolimus, with improved water solubility, to be a candidate mTORC1 inhibitor for human disc disease treatment.

## 5 CLINICAL RELEVANCE OF MTOR SIGNALING IN HUMAN DISC TISSUES

We examined the in vivo involvement of mTOR signaling in human disc NP surgical specimens. Western blotting showed the expression of mTOR, RAPTOR, RICTOR, p70/S6K, and Akt and the phosphorylation of mTOR, p70/S6K, and Akt in all patient samples with 19 to 81 in ages and 2 to 5 in degeneration grades.^80^, ^98^ Regression analysis demonstrated age‐dependent decreases in Akt expression and phosphorylation levels.^80^ Furthermore, mTOR, p70/S6K, and Akt expression and phosphorylation were increased in Pfirrmann^53^ grade‐3 discs.^98^ Despite the elevated interest in mTOR signaling,^42^ its involvement in disc degeneration remains largely unknown. The observed findings of mTOR‐signaling molecules associated with aging and degeneration severity potentially support mTOR involvement in disc health. However, due to variations among in vivo expression and phosphorylation for intracellular signaling molecules, future examinations with larger sample sizes are necessary to find conclusive evidence.

## 6 FUTURE CLINICAL APPLICATIONS OF AUTOPHAGY‐MODULATING AND/OR MTOR SIGNALING‐MODULATING THERAPIES FOR DEGENERATIVE DISC DISEASE

We found the in vivo involvement of mTOR signaling in human degenerative disc NP tissues from patients. Therefore, we propose gene therapy, targeting mTORC1/RAPTOR disruption, and pharmacological therapy, using mTORC1‐inhibiting temsirolimus, as future treatment strategies for degenerative disc disease. During gene therapy, safety is a primary concern. However, the disc NP is a unique, encapsulated, isolated structure that may be suitable for local delivery,^112^ and the prolonged >20‐week RNAi of a target gene, without adverse effects, has been reported.^113^ During drug therapy, local administration to the disc is preferable. The systemic administration of mTORC1 inhibitors is potentially risky due to serious adverse effects, including immunosuppression.^114^ Therefore, intradiscal gene and pharmacological therapies targeting mTORC1/RAPTOR suppression are potential biological treatments for degenerative disc disease. More specifically, based on by the consistent anti‐apoptotic, anti‐senescent, and anti‐catabolic effects of mTORC1/RAPTOR RNAi and mTORC1‐inhibiting temsirolimus administration, the application to prevent the chronic progression of disc degeneration, for example, by gene therapy, and also to treat the acute phase of painful discs, for example, by pharmaceuticals, would be suggested after careful animal testing and human clinical trials.

## 7 CONCLUSION

This brief review describes the current understanding of autophagy and mTOR signaling in the intervertebral disc. In vitro serum and nutrient deprivation reduced rabbit disc AF cell proliferation and metabolic activity, induced the dynamic activation of disc cellular autophagy, and increased apoptosis and senescence.^67^ The selective RNAi‐mediated mTORC1/RAPTOR suppression and the pharmacological mTORC1 inhibition were both protective against inflammation‐induced apoptosis, senescence, and matrix catabolism and were associated with Akt and autophagy induction in human disc NP cells.^80^, ^98^ These effects were primarily Akt phosphorylation‐dependent, resulting from negative feedback following mTORC1 suppression.^98^ Temsirolimus, a rapamycin derivative with improved water solubility, was the most effective among the tested mTORC1 inhibitors.^98^ However, the extensive interference of mTORC1/mTORC2/mTOR and mTORC2/RICTOR and the pharmacological inhibition of multiple mTOR complexes did not protect human disc NP cells against inflammation.^80^, ^98^ In vivo, mTOR‐signaling molecules showed marked expression and phosphorylation in human degenerated discs, which decreased with age.^80^, ^98^ A mechanistic understanding of autophagy and mTOR signaling provides a basis for the development of biological therapies to treat degenerative disc disease.

## CONFLICT OF INTEREST

The authors have no conflicts of interest to declare.

## AUTHOR CONTRIBUTIONS

T.Y. drafted the article. M.I., Y.K., R.K. and K.K. revised it critically for important intellectual content. All authors approved the final version to be published.
